# A retrospective study of a weight estimation model for hospitalized bedridden patients based on anthropometric parameters

**DOI:** 10.7717/peerj.20698

**Published:** 2026-02-09

**Authors:** Ai Luo, Zheng Tang, Xiaojia Xu, Guifen Guan, Zehang Hong, Dong Xiao, Jieyi Xu, Rongkui Wu, Zhuoqing Hu

**Affiliations:** 1 Department of Endocrinology, The Second Affiliated Hospital, Guangzhou Medical University, Guangzhou, China; 2 Department of Critical Care Medicine, The Second Affiliated Hospital, Guangzhou Medical University, Guangzhou, China

**Keywords:** Weight, Anthropometric Parameters, Estimation Model

## Abstract

**Objective:**

The aim of this study was to construct a weight estimation model for bedridden patients using anthropometric parameters that are readily obtainable during routine clinical care.

**Methods:**

A retrospective study was conducted involving 494 bedridden inpatients from the Department of Endocrinology of a tertiary general hospital (February 2023–February 2024). Weight was measured via a calibrated wheelchair scale. Anthropometric parameters (age, height, wrist, lower limb, waist, and hip circumferences) were measured in the supine position by trained researchers using standardized tools and specific anatomical landmarks. The estimation models were developed using stepwise regression.

**Results:**

The final models demonstrated excellent performance. The male model achieved an adjusted-R^2^ of 0.901 and Root Mean Square Error (RMSE) of 3.81 kg; the female model achieved an adjusted-R^2^ of 0.829 and RMSE of 3.81 kg. Bland-Altman analysis confirmed strong agreement between the actual and estimated weigh values, with a mean difference close to 0, no significant proportional bias, and most differences residing within the 95% limits of agreement.

**Conclusion:**

The developed models provide a reliable, cost-effective method for weight estimation in bedridden patients, using parameters that can be integrated into routine clinical assessments, offering a practical alternative to specialized equipment in resource-limited settings.

## Introduction

Weight is an extremely important observational indicator in clinical practice. Upon hospital admission, in addition to considering basic vital signs, biochemical test indicators, and imaging results, the patient’s baseline weight and weight changes during treatment are also important references (Zhou et al., 2003; Li & Liu, 2003). Weight is involved in the evaluation of nutritional status, determination of caloric needs, calculation of specific medication dosages, and assessment of clinical issues such as edema. For example, dialysis patients must have their weight measured before and after dialysis treatment to determine the dehydration amount for that session and to ensure the accuracy of dehydration during treatment, preventing issues such as blood pressure instability, dizziness, and cramps caused by over or under dehydration (James et al., 2017). For patients with diabetic ketoacidosis, weight is used to calculate insulin infusion rates and glucose concentrations when adjusting insulin administration based on blood glucose monitoring (Writing Group of Clinical Guidelines for the Prevention and Treatment of Elderly Diabetes Mellitus in China, 2022).

Currently, ordinary patients measure their weight using a scale or platform scale, known as the “conventional equipment measurement method”. However, critically ill patients, such as those who have undergone organ transplant surgery, suffered a stroke, or have fractures, often cannot use a scale or platform scale due to being bedridden and unable to move freely. Therefore, their weight must be estimated by doctors and nurses based on clinical experience, known as the “subjective assessment method”. Due to the varying clinical experience of each doctor and nurse, and the lack of objective measurement standards, this method often has significant deviations compared to equipment measurement methods, failing to obtain objective and accurate weight values. The measurement of weight in critically ill patients has always been a focus in the medical engineering field. Internationally, research on weight measurement methods and equipment for critically ill patients generally focuses on medical electric beds, but these medical electronic beds are expensive and cannot be widely used in primary hospitals. In China, researcher developed the first electronic weighing bed to solve the problem of critically ill patients only having the suspension method or flatbed moving method for weight measurement, which required patients to endure painful movements during measurement, increasing the risk of condition deterioration (Zhang, Sun & Wang, 2001). Due to clinical treatment needs, electronic weighing beds, weight weighing systems, weight measurement devices, pressure ulcer prevention air cushion beds with weighing capabilities, and supine position weight scales based on the suspension method have been designed and developed over the past 20 years (Zhu et al., 2003; Yu & Li, 2007; Liu et al., 2016; Fan, Zhu & Xi, 2020). However, they all have issues such as large error ranges, requiring multiple medical staff to operate collaboratively to ensure patient safety, and being bulky. In summary, there are relatively few mature products available on the market both domestically and internationally, and the products that are available are generally expensive and difficult to popularize in primary hospitals.

Furthermore, various alternative weight estimation methods have been explored. Studies have found that the most accurate weight estimation methods are the 3D imaging system, patient self-estimation, the Lorenz formula, and family estimation; however, these methods have significant limitations in emergency settings (Wells et al., 2024). Patient self-estimation is considered the best option, but when patients cannot provide an estimate, alternative methods are needed. Single-variable anthropometric formulas, such as those using upper arm circumference, have low accuracy. The application of multivariable formulas for weight estimation in adults requires further research. Based on the above reasons, the primary aim of this study was to develop and validate gender-specific weight estimation models for bedridden inpatients using a set of easily obtainable anthropometric parameters. We hypothesized that: (1) a combination of basic anthropometric measurements (including limb, waist, and hip circumferences) would strongly correlate with and accurately estimate actual body weight; and (2) estimation models stratified by gender would demonstrate superior accuracy and lower estimation error compared to a single generic model, will be highly valuable.

## Subjects and Methods

### Subjects

A total of 494 patients hospitalized in the Department of Endocrinology from February 2023 to February 2024 were collected. The target population for this study was adult, bedridden inpatients requiring accurate weight assessment for clinical management. Patients from the Department of Endocrinology were chosen as a representative sample of this population because they commonly present with conditions (*e.g.*, complicated diabetes, severe infections) that often lead to prolonged bed rest and a direct clinical need for precise weight monitoring.

Inclusion criteria: (1) hospitalized patients aged ≥18 years; (2) bedridden patients unable to stand for weight measurement. Exclusion criteria: (1) pregnant or lactating patients; (2) patients with edema were excluded because fluid retention causes rapid, non-physiological fluctuations in body measurements, which do not represent the stable body composition underlying our model; (3) patients with amputations or severe physical deformities that would prevent accurate anthropometric measurement; (4) patients with severely missing or incomplete data. This study was approved by the hospital’s ethics committee (2024-hg-ks-29). Since this was a retrospective study and did not collect sensitive or personal privacy information, the requirement for informed consent was exempted, which was also supported by the ethics committee.

According to epidemiological research requirements, each factor included needs 5-10 samples for validation (Shipe et al., 2019). Based on literature review and expert consultation, seven relevant factors were proposed. Considering the number of independent variables in the regression model, model fit, and significance level, the required sample size was determined using power analysis in multiple linear regression. The sample size calculation formula is as follows: $n= \frac{2\cdot k}{1-{R}^{2}} \cdot \frac{1+k}{1-\alpha } $.

With seven independent variables, an expected model fit of 0.85, and a significance level of 0.05, the minimum sample size required was 424 cases.

### Instruments and methods

Weight was measured using a calibrated weight measurement instrument (model: RGZ-120-RT). This instrument was calibrated annually by the manufacturer’s metrology service, and a zero-point check was performed before each measurement session. Since patients could not stand, weight was measured in a wheelchair, and the wheelchair weight was subtracted to obtain the actual patient weight. Since patients could not stand, the measurement procedure was as follows: (1) The empty wheelchair was weighed, and the scale was tared to zero. (2) The patient was carefully transferred into the wheelchair by trained nursing staff. (3) The total weight (patient + wheelchair) was recorded. (4) The patient’s weight was obtained by subtracting the pre-measured weight of the wheelchair. Each patient’s weight was measured twice, and the average value was used for analysis if the difference was less than 0.5 kg.

All anthropometric parameters were measured in the supine position by trained researchers using a standardized medical health ruler (Model: LTBMI150) to ensure consistency. The specific measurement protocols were as follows: wrist circumference was measured at the narrowest part of the non-dominant wrist, distal to the styloid processes of the ulna and radius; lower limb circumference was measured two cm above the level of the medial and lateral malleoli of the non-dominant side; waist circumference at the midpoint between the lower rib margin and the iliac crest; hip circumference at the level of the greatest protrusion of the gluteal muscles; and height was obtained using a non-stretchable tape measure from the vertex of the head to the heel while the patient maintained a fully extended supine position.

### Statistical methods

Statistical analyses were performed using SPSS 26.0 and R (4.4.0; R Core Team, 2024). Data normality was assessed with the Kolmogorov–Smirnov test and probability-probability plots. Normally distributed data are presented as mean ± standard deviation and compared using independent sample t-tests; non-normally distributed data were compared using the Wilcoxon rank-sum test. Correlations were analyzed with Pearson or Spearman coefficients.

The estimation model was developed *via* stepwise regression and generalized linear model fitting. To mitigate overfitting, model performance was evaluated with 10-fold cross-validation. Multicollinearity was controlled by retaining only variables with a Variance Inflation Factor (VIF) < 5.0. Agreement between estimated and measured weights was assessed using a paired *t*-test for systematic bias, Bland-Altman analysis for limits of agreement, and the Two One-Sided Tests (TOST) procedure with a ±5 kg clinical margin for equivalence.

## Results

### General information and measurement results

The age, height, weight, wrist circumference, lower limb circumference, waist circumference, and hip circumference of the subjects are shown in Table 1. Except for age and hip circumference, height, weight, wrist circumference, lower limb circumference, and waist circumference showed statistical differences between genders (*P* < 0.05).

**Table 1:** General information and measurement results of subjects.

Variable	Overall (*n* = 494)	Female (*n* = 248)	Male (*n* = 246)	*P*-value
Age (years)	61.32 ± 14.14	62.11 ± 14.67	60.53 ± 13.57	0.09^a^
Height (cm)	161.67 ± 8.78	155.50 ± 6.21	167.90 ± 6.22	<0.001^a^
Weigh (Kg)	63.43 ± 12.43	58.79 ± 10.75	68.12 ± 12.28	<0.001^a^
Wrist circumference (cm)	15.75 ± 1.31	15.09 ± 1.13	16.41 ± 1.12	<0.001^a^
Lower limb circumference (cm)	20.47 ± 1.84	19.93 ± 1.87	21.01 ± 1.65	<0.001^b^
Waist circumference (cm)	85.24 ± 9.79	83.85 ± 9.85	86.64 ± 9.56	<0.001^b^
Hip circumference (cm)	92.06 ± 7.21	91.91 ± 7.46	92.22 ± 6.96	0.49^a^

**Notes.**

a Wilcoxon test for two independent samples.

b Independent sample *t*-test.

### Variable correlation and multicollinearity assessment

All anthropometric parameters demonstrated statistically significant correlations with body weight (*p* < 0.05), except for age in females. Hip circumference exhibited the strongest correlation (Pearson *r* = 0.85 in females, *r* = 0.86 in males), followed by waist circumference (*r* = 0.82 in females, *r* = 0.88 in males) and lower limb circumference (*r* = 0.66 in females, *r* = 0.72 in males), which also showed strong positive correlations. Wrist circumference demonstrated moderate positive correlations (*r* = 0.58 in females, *r* = 0.61 in males), while height was moderately positively correlated (*r* = 0.39 in females, *r* = 0.46 in males). Age showed a significant but weak negative correlation in males (r = −0.27), whereas its correlation was non-significant in females (*r* = 0.02). The Spearman correlation analysis yielded consistent results.

Despite observing high correlations between some predictors and the outcome (weight), a subsequent VIF analysis confirmed that multicollinearity among the predictors themselves was not severe. All variables retained in the final models had VIF values below the conservative threshold of 5.0 (the highest being 4.4 for waist circumference in the male model). This indicates that while the variables are strong individual predictors of weight, they provide sufficiently independent information to be included together in a stable multiple regression model (Table 2).

**Table 2:** Correlation and multicollinearity analysis results of various variables with weight.

Variable	Correlation	Correlation	VIF	VIF
	(Female)	(Male)	(Female)	(Male)
Age	0.02	−0.27	1.20	1.20
Height	0.39	0.46	1.10	1.40
Wrist circumference	0.58	0.61	1.50	1.60
Lower limb circumference	0.66	0.72	1.90	2.00
Waist circumference	0.82	0.88	4.10	4.40
Hip circumference	0.85	0.86	4.30	4.10

### Coefficient test results and regression equations of the model

Using cross-validation parameters set to 10, stepwise regression was used to select variables, and the model was fitted using a generalized linear model with Identity as the link function. The results are shown in Table 3. The following equations were derived:

\begin{eqnarray*}\mathrm{Weight}~(\mathrm{Female})=-92.931-0.097\times \mathrm{Age}+0.273\times \mathrm{Height}\nonumber\\\displaystyle +~0.946\times \mathrm{Wrist}~\mathrm{circumference}+0.559\times \text{Lower Limb Circumference}+0.394\nonumber\\\displaystyle \times \text{Waist Circumference}+0.620\times \text{Hip Circumference};\nonumber\\\displaystyle \mathrm{Weight}~(\mathrm{Male})=-104.873-0.105\times \mathrm{Age}+0.297\times \mathrm{Height}+0.508\times \text{Wrist circumference}\nonumber\\\displaystyle +~1.149\times \text{Lower Limb Circumference}+0.653\times \text{Waist Circumference}\nonumber\\\displaystyle +~0.439\times \text{Hip Circumference}. \end{eqnarray*}

**Table 3:** Coefficient test results.

Item	β-value	Standard error	*t*-Value	*P*-value
**Female**				
Constant	−92.93	9.11	−10.20	<0.01
Age	−0.10	0.03	−3.70	<0.01
Height	0.27	0.06	4.60	<0.01
Wrist circumference	0.95	0.37	2.58	0.01
Lower limb	0.559	0.24	2.33	0.02
Circumference				
Waist circumference	0.39	0.07	5.95	<0.01
Hip circumference	0.62	0.09	7.04	<0.01
**Male**				
Constant	−104.87	9.04	−11.60	<0.01
Age	−0.11	0.02	−4.72	<0.01
Height	0.30	0.05	5.48	<0.01
Wrist circumference	0.51	0.32	1.57	0.12
Lower limb	1.15	0.26	4.48	<0.01
Circumference				
Waist circumference	0.65	0.07	9.49	<0.01
Hip circumference	0.44	0.10	4.51	<0.01

### Model fit and estimation performance results

**Figure 1:**
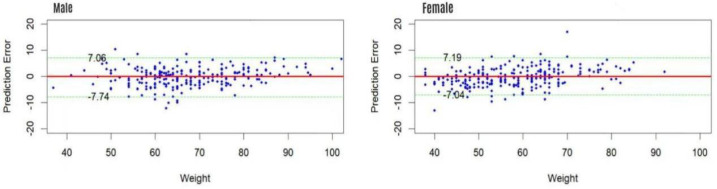
The Bland-Altman plot of actual weigh and estimated weigh.

The gender-specific estimation models demonstrated excellent fit and estimative accuracy (Table 4). The agreement between measured and estimated weights was rigorously assessed through Bland-Altman analysis, equivalence testing, and an examination of proportional bias, with key results summarized in Table 5. Briefly, the mean differences were small (0.13 kg for males, 0.45 kg for females) and the 95% limits of agreement were clinically acceptable (Fig. 1). Critically, equivalence testing using a ±5 kg margin confirmed that the estimation method is clinically equivalent to direct measurement for both genders (*p* < 0.001). Furthermore, the analysis revealed no significant proportional bias (*p* = 0.64 for males; *p* = 0.97 for females).

**Table 4:** Comparison of estimation performance of different models.

Gender	AIC	R^2^	Adjusted-R^2^	MSE	RMSE
Male	123.45	0.90	0.90	14.56	3.81
Female	130.67	0.83	0.83	15.23	3.81

**Table 5:** Agreement, equivalence, and bias analysis between measured and estimated weight.

Analysis metric	Male model	Female model
**Bland–Altman analysis**		
Mean difference (kg)	0.13	0.45
95% Limits of agreement (kg)	−7.74∼7.74	−7.04∼7.19
**Equivalence testing (TOST)**		
Clinical margin (kg)	±5	±5
90% CI of mean difference (kg)	(−0.25, 0.52)	(−0.12, 1.02)
*P*-value for equivalence	<0.001	<0.001
**Proportional bias analysis**		
Regression slope (difference *vs* average)	0.04	0.09
*P*-value for slope	0.64	0.97

## Discussion

Weight estimation is crucial in emergency medical care, but direct weight measurement is often challenging due to various constraints. Inaccurate weight estimation can lead to incorrect medication dosages, thereby endangering patient safety. Moreover, the field lacks standardized criteria for determining the accuracy and clinical acceptability of weight estimation methods, and no other estimation tools have been specifically developed and validated for direct comparison in the bedridden patient population (Wells et al., 2024). In contrast to previously reported methods that rely on expensive equipment or subjective estimation, this acknowledges the existence of alternative methods (*e.g.*, bioelectrical impedance analysis) whose primary drawback is the frequent requirement for costly, specialized equipment (Alijanian et al., 2012). This study successfully constructed a highly accurate, gender-specific estimation model for bedridden patients based on easily obtainable anthropometric parameters. The required tools are ubiquitous, the procedure can be rapidly integrated into routine nursing care with minimal training, and it provides a immediately actionable weight estimate. For the purpose of generating a practical weight estimate in resource-aware clinical settings.

Recent studies (Pu et al., 2021) have demonstrated the correlation between hip circumference and the weight of children and adolescents using linear regression equations, providing a clinically accurate and simple weight estimation method based on hip circumference for most children and adolescents (Wang, 2023). Some researchers have used height, waist circumference, chest circumference, and upper arm circumference to estimate the weight of 263 physical examination subjects, achieving a good model fit, although it has not yet been validated (Ye, Wu & Han, 2012). Building upon the foundation of anthropometric estimation, this study developed and validated a model specifically for bedridden inpatients using six explanatory variables (age, height, wrist, lower limb, waist, and hip circumferences). Our initial analyses confirmed significant gender-based differences in these parameters and their relationship with weight, prompting an investigation into gender-specific modeling. The results strongly justified this approach: the separate models demonstrated superior performance, with the male model achieving an adjusted R^2^ of 0.901 and RMSE of 3.81 kg, and the female model an adjusted R^2^ of 0.829 and RMSE of 3.81 kg. Although the male model showed a slightly better fit (*e.g.*, lower AIC, higher R-squared), the RMSE was virtually identical for both genders. This indicates that while the male model explained a greater proportion of the variance in our sample, both models attained a similarly high and clinically useful level of estimation accuracy for their respective populations.

The selection of a focused set of anthropometric dimensions—specifically limb, waist, and hip circumferences—was a deliberate strategy grounded in clinical practicality, scientific rationale, and the pursuit of a transparent, deployable model. These sites were chosen primarily for their high feasibility in routine nursing practice; they are easily accessible, can be measured standardizedly in the supine position with minimal patient discomfort, and require no specialized equipment. Scientifically, this combination captures both peripheral and central body composition, which are known to correlate strongly with total body mass. This parsimonious approach, which avoids an excessively complex set of measurements, was sufficient to achieve excellent estimative accuracy. Furthermore, it aligns perfectly with our choice of multiple linear regression, a method that generates intuitive, linear equations, ensuring the model remains interpretable and easy for clinicians to apply at the bedside—a key advantage over “black-box” machine learning algorithms (Christodoulou et al., 2019). The excellent performance of our gender-stratified models demonstrates that this combination of carefully selected inputs and a robust, interpretable modeling technique fulfills the core objective of creating a reliable and readily implementable tool for bedridden patients.

The consistent performance during 10-fold cross-validation, which attests to the model’s robustness and lack of overfitting, supports the high internal validity and potential applicability of our estimation models to their intended clinical context—adult, non-edematous, bedridden inpatients. The models are particularly suited for use in departments like endocrinology wards where patients require accurate weight for drug dosing or nutritional support but lack access to specialized weighing equipment. The requirement for simple anthropometric measurements makes these models highly feasible for nurses and healthcare workers to implement during routine patient care.

However, several points should be considered for application. First, the model currently exists as a statistical equation and has not been transformed into a user-friendly interface or tool, which may limit its practicality; future development of a web-based calculator is planned to address this. Second, the data were sourced from a single hospital, and external validation across different institutions is necessary to confirm generalizability. Third, this model requires validation in other specific case groups, and further research with larger sample sizes and alternative modeling techniques is warranted to refine its performance.

## Implications of the Findings

This study provides a scientifically-grounded model to replace subjective weight estimation in bedridden patients, utilizing simple anthropometric measures integral to routine nursing care. This approach offers a feasible, cost-effective, and equitable solution, particularly for resource-limited settings, as it requires no specialized equipment and uses gender-specific equations to account for biological differences. The primary significance of our work lies in establishing compelling preliminary evidence that a core set of anthropometric parameters can form the basis for accurate weight estimation, setting a foundation for future validation and broader application.

## Conclusion

In summary, the estimation models established separately for different genders showed slight differences in performance but overall performed well. The weight estimated models provided in this study can serve as a reference for weight measurement of bedridden patients who cannot get out of bed for various reasons, providing a simple, low-cost, and easy-to-implement solution for clinical healthcare professionals. This model has the advantages of being simple to operate, minimally invasive to patients, unaffected by the environment, and low-cost, making it easy to promote and implement, and it holds significant practical value for assisting clinical treatment.

## Supplemental Information

10.7717/peerj.20698/supp-1 Supplemental Information 1 Raw Data

## Additional Information and Declarations

### Data Availability

The following information was supplied regarding data availability: The raw data are available in the Supplemental File.
